# Response of Roses (*Rosa hybrida* L. ‘Herbert Stevens’) to Foliar Application of Polyamines on Root Development, Flowering, Photosynthetic Pigments, Antioxidant Enzymes Activity and NPK

**DOI:** 10.1038/s41598-019-52547-1

**Published:** 2019-11-05

**Authors:** Fereshteh Yousefi, Zohreh Jabbarzadeh, Jafar Amiri, Mir Hassan Rasouli-Sadaghiani

**Affiliations:** 1 Department of Horticultural Science, Faculty of Agriculture, Urmia University, Urmia, Iran; 2 Department of Soil Science, Faculty of Agriculture, Urmia University, Urmia, Iran

**Keywords:** Photosynthesis, Plant physiology

## Abstract

The effect of foliar application of polyamines on roses (*Rosa hybrida* cv. ‘Herbert Stevens’) was investigated in a factorial experiment based on a completely randomized design with three replications in a greenhouse. Two factors were applied including polyamine type (putrescine, spermidine, and spermine) and polyamine concentration (0, 1, 2 and 4 mM). The recorded traits included root fresh and dry weight, root length, number of flowers, flower longevity, chlorophyll content, carotenoids, antioxidant enzymes activity (catalase, ascorbate peroxidase and guaiacol peroxidase) and some macronutrients such as nitrogen, phosphorus and potassium. The results showed that among polyamines, putrescine had the greatest effect on root dry weight; spermidine showed the greatest effect on root length, chlorophyll content, plant phosphorus and spermine affected root fresh weight and flower longevity most strongly. Polyamine concentration of 1 mM had the strongest effect on flower longevity, carotenoids, nitrogen and phosphorus content. The highest potassium rate was observed in treatments with the concentration of 4 mM. Polyamine treatments had no significant effect on the number of flowers per plant and antioxidant enzymes.

## Introduction

Roses are originated from the northern hemisphere and are mainly grown in temperate zones^1^. In fact, the earliest roses appeared in states of Colorado and Oregon around 30 million years ago^2^. Due to its diverse growth habits, excellent appearance, variety in size and type, attractive colors, delicious aromas and countless cultivars, roses are highly appreciated^3^. This plant can be identified by its bushy and shrubby appearance with straight woody branches, somewhat covered with prickles^2^. They usually grow in two forms: growing up in the form of shrubs or creeping over surfaces^4^.

Polyamines are aliphatic hydrocarbons with low molecular weight and two amino groups at their ends^5^. Diamines putrescine, triamine spermidine, and tetraamines spermine are the most commonly found polyamines in plants^6^. Polyamines can play roles in seed germination, the breakdown of tuber dormancy, rooting control, flower development and initiation, the postponement of aging, organogenesis, and the response to biotic and abiotic stresses. Polyamines also act as regulatory molecules in basic cellular processes, including cell division, differentiation, gene expression, and DNA and RNA synthesis^7^. All eukaryotic cells contain significant amounts of polyamines, such as spermine, spermidine, and putrescine precursor^8^.

Putrescine spray on *Antirrhinum majus* L. showed that putrescine at the rate of 200 mg/l had a significant effect on root length and root fresh and dry weight^9^. The effect of putrescine and growth medium on vegetative growth and chemical components of *Populus* × *euramericana* L. showed that the culture medium containing a mixture of sand and clay with putrescine spray at the rate of 50 mg/l had the greatest effect on length, diameter, and fresh and dry weight of roots^10^. Results of a study on the effect of polyamines on Basil plants showed that application of polyamines (putrescine, spermidine and spermine) increased root fresh and dry weight in comparison with the control plants^11^. Foliar application of polyamines on roses showed that all three polyamines, i.e. putrescine, spermidine and spermine, increased vase life, flower bud diameter, and flower bud length compared to the control^12^. Pre- and post-harvest application of putrescine on Alstroemeria extended vase life, and the best results were observed when putrescine was used before and after harvest^13^. The application of putrescine and humic acid in roses showed that putrescine treatment with humic acid increased the vase life of flowers as compared to the control^14^. Results of an experiment on the effect of different polyamines on *Dianthus caryophyllus* L. showed the highest chlorophyll content in spermidine treatments. Also, the highest reduction in the amount of ethylene was observed in the treatment with spermidine^15^. Another study on the effect of putrescine and uniconazole on some characteristics of *Salvia splendens* Sellow ex Roem. & Schult indicated that putrescine at the concentration of 150 mg/l had the strongest effect on chlorophyll and carotenoids^16^. The application of putrescine and alpha-tocopherol enhanced the content of chlorophyll and carotenoids (leaf) significantly compared to the control^17^. Spraying of Alstroemeria with putrescine and spermine in two stages, i.e. before and after harvest, increased the activity of catalase enzyme. Putrescine and spermine reduced the activity of leaf chlorophyllase enzymes and flower pectinase and phenoloxidase^18^. In another study focused on the effect of exogenous spermidine and spermine on antioxidant metabolism associated with cold-induced leaf senescence in Zoysia grass (*Zoysia japonica* Steud.) results showed that application of spermine and spermidine enhanced the activities of peroxidase, catalase and ascorbate peroxidase under cold acclimation conditions; however, they had no effect on catalase, ascorbate peroxidase and peroxidase activity under normal conditions^19^. The effect of putrescine on *Gladiolus* L. spp. showed that among three concentrations of putrescine, the concentration of 200 mg/l had the greatest effect on increasing such nutrients as nitrogen, phosphorus, and potassium^20^. Spraying rose plants with putrescine and humic acid showed that putrescine + humic acid treatment increased the content of nitrogen, phosphorus, potassium, magnesium, and calcium in the leaves^14^.

The aim of this experiment was to investigate the effects of various concentrations of putrescine, spermidine, and spermine on some morphological and physiological characteristics, antioxidant enzyme activities and leaf elements in non-stress conditions on rose cv. ‘Herbert Stevens’.

## Materials and Methods

### Plant materials and treatments

In order to investigate the effect of foliar application of polyamines on some morphological and physiological characteristics of roses ‘Herbert Stevens’, a factorial experiment was conducted in a completely randomized design with two factors including polyamine type (putrescine, spermidine, and spermine) and different concentrations of polyamines (0 as control, 1, 2, and 4 mM) with three replications. Each replication included two pots and each pot was composed of one single plant. This research was carried out on rose plants for 2 months during which the plants were subjected to the foliar application in 2-week intervals. Plastic pots with a diameter of 14 cm and a height of 11 cm were used. The greenhouse day/night temperature was set at 28–30/20–23 °C. The cuttings of roses were first planted in a sand bed to bear roots. Then, they were transferred to separate pots and were kept there for two months to fully develop new plants. After establishment, the plants were prepared for spraying. The soil used in the pots was composed of garden soil and sand (3:1 v/v). In order to analyze the soil of the pots, a combined soil sample was sent to the soil analysis laboratory in Urmia University’s Soil Department. The results are presented in Table 1. Three types of polyamines, including putrescine, spermidine, and spermine, were purchased from Sigma Company. The foliar application was performed in four steps at the intervals of 15 days in the morning. The polyamines were dissolved in water at each treatment step. During the study, no nutrient solution was used for irrigation.

**Table 1:** Results of soil analysis.

Texture	Silt %	Sand %	Clay %	pH	EC ds/m	O–M %	C–O %	CaCO_3_ %	P mg/kg	K mg/kg
Sandy clay loamy/Sandy loamy	20	60	20	7.2	1.4	1.62	0.94	8.5	28.04	307

C–O: Organic carbon, O–M: Organic matter.

### Growth characteristics

Two weeks after the final treatment, the morphological parameters were measured. The root length was precisely measured by a ruler, after being washed by water and a digital balance (0.00001) was used to measure root fresh and dry weight. To evaluate the longevity of the flowers per plant, the days were counted from anthesis until when the flowers could keep their freshness. The number of flowers per plant was also counted.

### Photosynthesis pigments

The amount of photosynthesis pigments including chlorophyll (*a*, *b* and total) and carotenoids were determined according to the method of Gross^21^. Therefore, acetone 80% was used for leaf chlorophyll extraction (each sample was 0.25 g) and then extracts were centrifuged (3000 rpm) for 10 minutes. The absorbance of extracts was read with a spectrophotometer (at 470, 645 and 663 nm). Finally, chlorophyll content and carotenoids were determined by following equations:

Chlorophyll *a* (mgl^−1^) = 12.7*A*_663_ $${-}$$ 2.69*A*_645_

Chlorophyll *b* (mgl^−1^) = 22.9*A*_645_ $${-}$$ 4.68*A*_663_

Total chlorophyll = Chlorophyll *a* + Chlorophyll *b*

Carotenoids = (1000*A*_470_ ${-}$ 1.43 Chl *a* ${-}$35.87 Chl *b*)/205

### Antioxidant enzymes activity

The Kang and Saltiveit’s^22^ method was used to provide plant extracts to determine the activity of catalase, ascorbate peroxidase and guaiacol peroxidase. Frozen leaf tissues (0.5 g) were homogenized with buffer solution (pH = 7.5) containing 0.05 mM hydrochloric acid, 3 M magnesium chloride and 1 M EDTA. The homogenate was then centrifuged at 4000 rpm for 20 minutes at 4 °C. Among the antioxidant enzymes, activity of catalase was measured by Aebi’s^23^ method. The reaction mixture consisted of 50 mM phosphate buffer (pH = 7.5), hydrogen peroxide (1%) and 0.3 ml extract. The absorbance was read at 240 nm. The activity of ascorbate peroxidase was evaluated by Nakano and Asada’s^24^ method. 50 mM phosphate buffer (containing 0.1 mM EDTA and 1 mM Sodium ascorbate) and hydrogen peroxide (1%) were added to 0.1 ml extract. The change in the absorbance was read at 290 nm. Then, the activity of guaiacol peroxidase enzyme was calculated by increasing the absorbance over a minute at 420 nm using a spectrophotometer. The activity of guaiacol peroxidase was found out by Updhyaya *et al*.’s^25^ method at 420 nm. In brief, reaction mixture consisted of 2.5 ml of phosphate buffer (50 mM), 1 ml guaiacol (1%), 1 ml hydrogen peroxide (1%) and 0.1 ml enzyme extract.

### Leaf minerals

For this purpose, the leaf samples of the treated plants were washed and then put in the oven at 70 °C for 72 hours. The oven dried samples were powdered. The nitrogen content of plant leaves was measured by kjeldahl method according to Ohayama *et al*.’s^26^ protocol. The Kjeldahl procedure involves three major steps: the digestion (organic nitrogen is converted into ${{\rm{NH}}}_{4}^{+}$), the distillation and the titration. In order to measure potassium and phosphorus content, the extract was prepared by dry digestion of samples. The amount of plant phosphorus was estimated by the colorimetric method according to Ohayama *et al*.’s^26^. For this purpose, Ammonium vanadate and ammonium molybdate solution and phosphorus standards were prepared. Samples were read with spectrophotometer at 470 nm and after calculating, the amount of phosphorus was expressed as percentage. The potassium content of the plants was measured by using a flame photometer with Ohayama *et al*.’s^26^ method.

Finally, the SAS software package (version 9.1) was employed for data analysis, and the means of the tested traits were compared using Tukey’s Test at the *p* < 0.01 and *p* < 0.05 levels.

## Results and Discussions

### Root length and fresh and dry weight of root

Analysis of variance results showed that the effect of polyamine sources on root fresh weight and flower longevity were significant at 1% level, the effect of polyamine concentrations on root length, root fresh and dry weight and flower longevity at 1% level and their interaction effects on root length, root fresh and dry weight was significant at 1% level (Table 2).

**Table 2:** Results of variance analysis of the effect of polyamines sources and concentrations on some morphological characteristics of rose ‘Herbert Stevens’.

Changes sources	DF	Mean squares				
		Root Length	Root Fresh Weigth	Root Dry Weigth	Flower Number	Flower Longevity
Polyamin source	2	2.09^†ns^	25.25^**^	0.98^ns^	0.58^ns^	8.77^*^
Polyamin concentration	3	28.13^**^	16.78^**^	4.36^**^	0.32^ns^	40.18^**^
Source*concentration	6	27.10^**^	19.73^**^	2.86^**^	0.65^ns^	1.96^ns^
Error	24	4.81	3.03	0.77	0.44	2.27
CV (%)		12.79	14.85	20.26	38.09	14.52

^†^ns, * and ** respectively non-significant, significant at 5 and 1 percent.

According to the results of the means comparison for root length (Table 3), only spermidine (2 mM) and putrescine (1 mM) showed significant differences with the control, but there were no significant differences in other treatments with the control. The highest root length (23 cm) was related to spermidine treatment at the concentration of 2 mM which had no significant difference with that of the plants treated with 1 mM putrescine. The lowest root length (14 cm) was observed in plants treated with 1 mM spermidine. According to Table 2, only the treatments of spermine (4 mM) and spermidine (4 mM) induced a significant difference in root fresh weight with control plants. The highest fresh weight (15.74 g) was related to spermine treatment at the concentration of 4 mM, and the lowest fresh weight (5.82 g) was associated with spermidine treatment at the concentration of 4 mM. However, other treatments of polyamines showed no significant difference with the control in root fresh weight. Also, the results of the means comparison of the interaction between the type and concentration of polyamines for root dry weight (Table 3) showed insignificant differences between the control and polyamine treatments. According to Table 3, the highest root dry weight (6.06 g) was obtained from spermidine treatment at the concentration of 2 mM and the lowest dry weight (2.36 g) was related to spermidine at the concentration of 4 mM.

**Table 3:** The effect of putrescine (Put), spermidine (Spd) and spermine (Spm) on root length, root fresh and dry weight and chlorophyll content of rose ‘Herbert Stevens’

Treatment	Root length (cm)	Root fresh weight (g)	Root dry weight (g)	Chlorophyll *a* (mg/g fw)	Chlorophyll *b* (mg/g fw)	Total Chlorophyll (mg/g fw)
Control	14.66^b^	10.18^ab^	4^ab^	0.93^c^	0.29^bcd^	1.22^de^
Put 1 mM	21.33^ab^	12.36^a^	4.69^ab^	1.04^bc^	0.18^cd^	1.23^de^
Put 2 mM	17^ab^	14.07^a^	5.92^a^	1.24^bc^	0.37^abc^	1.62^cd^
Put 4 mM	17.5^ab^	11.85^ab^	3.93^ab^	1.19^bc^	0.24^cd^	1.44^cde^
Spd 1 mM	14^b^	10.73^ab^	3.83^ab^	1.34^bc^	0.38^abc^	1.72^bc^
Spd 2 mM	23^a^	13.74^a^	6.06^a^	1.23^bc^	0.38^abc^	1.61^cd^
Spd 4 mM	16.33^ab^	5.82^b^	2.36^b^	1.88^a^	0.58^a^	2.46^a^
Spm 1 mM	18.33^ab^	13.66^a^	4.31^ab^	2.01^a^	0.05^d^	2.06^ab^
Spm 2 mM	16.33^ab^	12.18^ab^	3.99^ab^	1.16^bc^	0.52^ab^	1.68^bc^
Spm 4 mM	18^ab^	15.74^a^	4.92^a^	0.97^bc^	0.21^cd^	1.18^e^

In each column, values followed by the same letter(s) do not differ significantly at $\propto$ = 0.01.

Reports indicate that free polyamines such as agmatine, putrescine, spermidine, and spermine and connecting polyamines such as caffeoyl putrescine and feruloyl putrescine have been observed in root tissue like insoluble binding polyamines^27^. Polyamines are a precursor to many alkaloids, some of which play a role in root development. For example, polyamines in Asteraceae species produce alkaloids like pyrrolizidine whose synthesis is unique to the root and appears to be related to root growth as the reduction or stopping of its synthesis results in stopping root growth^28^. On the other hand, root growth and differentiation in plants are closely related to plant hormones. Auxin is one of the most widely used plant hormones^29^. Indole-3-acetic acid is the main auxin present in many plants and is responsible for the structure of the root system and development stages of root^30^. Indole-3-butyric acid is also another plant auxin that effectively promotes the development of adventitious roots. In plants, root development during biosynthesis, transmission, and signaling of auxins reaches the maximum. Other plant hormones like cytokinin, brassinosteroids, ethylene, abscisic acid, gibberellins, jasmonic acid, polyamines, and strigolactones are also involved in these processes. Polyamines and brassinosteroids positively regulate the lateral roots; likewise, polyamines, brassinosteroids, and strigolactones positively regulate the development of primary roots. By increasing our knowledge of the roots, it has been shown that auxin acts as a central axis and other plant hormones communicate with it to regulate root development^29^. The relationship between polyamines and auxin in root formation and its growth in two orange cultivars was investigated and it was observed that the addition of polyamines to the induction medium containing auxin (NAA and IBA) significantly improved root development and growth, whilst these processes were stopped in the presence of biosynthesis inhibitors of polyamines, such as DFMO^31^.

The role of polyamines in increasing cell division, their presence in roots, biosynthesis of many alkaloids, the positive interaction of polyamines with auxins, and the observations about the use of biosynthesis inhibitors of polyamines suggest that polyamines play a role in controlling rooting and root system expansion, so the increased root production and root lengthening enhance root fresh and dry weight. As was seen in the present study, the treatment of polyamines increased root length, but this increase in root length was significant only in the treatments of 1 mM putrescine and 2 mM spermidine and 1 and 4 mM spermine. In this research, there were not any significant differences in increasing root length of the plants, and this may be related to the fact that the roots were cut off during their separation from the soil. Regarding root fresh weight, only significant differences were observed in fresh weight gain in spermine treatment (4 mM), while none of the polyamines and their concentrations showed significant differences in root dry weight compared to the control. Possibly, the concentrations used in this study did not significantly affect root growth and the fresh and dry weight of the roots. The positive effects of putrescine on root growth have been reported in other studies. Putrescine treatment on *Antirrhinum* spp. and *Populus* showed that all concentrations of putrescine increased root length and fresh and dry weight of roots when compared to the control^9,10^. In *Bougainvillea glabra* Choicy, putrescine application increased the fresh and dry weight of roots^32^. In strawberries, the foliar treatment of bushes with putrescine increased root length versus the control^33^. In another study on pepper (*Capsicum annum* L.) seedling, it was reported that among different polyamine treatments, only putrescine (2.5 mM) significantly increased the root fresh and dry weight under salt stress (100 mM NaCl), but there was not any significant difference in root fresh and dry weight between the control and treated plant on normal conditions^34^.

### Number of flowers and flower longevity

According to the results of means comparison, the number of flowers per plant was not affected by polyamine treatments. Studies on polyamines show that they have different effects on plants, even across the cultivars of a species. In strawberries, putrescine treatment did not show a significant increase in flower number compared to the control^33^. In *Antirrhinum* spp., all concentrations of putrescine treatment increased the number of inflorescences^9^. However, there was no significant effect of putrescine treatment on the number of flowers in *Calendula officinalis* L.^35^. According to the results of the above studies, it is observed that polyamines have different effects on the number of flowers in different plants and even the cultivars of a species. As in the present study, there was no significant effect on the number of flowers in rose “Herbert Stevens”.

**Figure 1:**
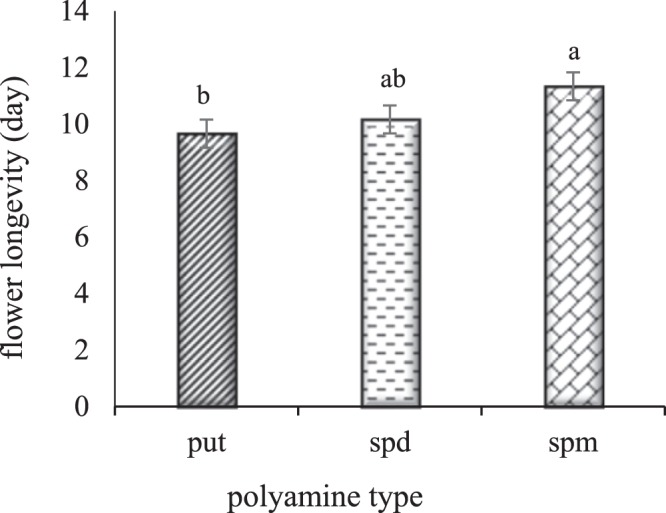
The effect of polyamine type on flower longevity of rose ‘Herbert Stevens’. Values followed by the same letter(s) do not differ significantly at $\propto$ = 0.01.

**Figure 2:**
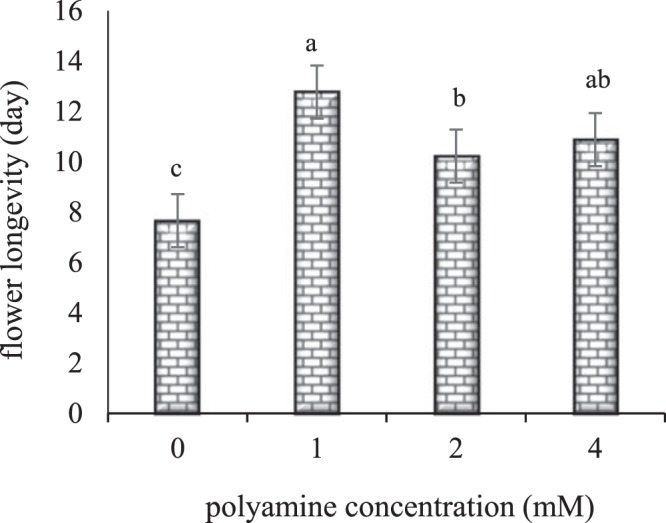
The effect of polyamine concentration on flower longevity of rose ‘Herbert Stevens’. Values followed by the same letter(s) do not differ significantly at $\propto$ = 0.01.

According to means comparison (Fig. 1), the highest flower longevity per plant (11.33 days) was related to spermine treatment and the lowest flower longevity (9.66 days) was related to putrescine treatment. However, no significant difference was observed between spermine and spermidine treatments and also there were not any significant differences between putrescine and spermidine treatment. On the other hand, the results of different concentrations of polyamines (Fig. 2) showed that all treatments of polyamines exhibited significant differences with the control plants. The highest flower longevity (12.77 days) was observed at the treatment with the concentration of 1 mM and the lowest flower longevity (7.66 days) was related to the control. No significant difference was observed between treatments with the concentrations of 1 mM and 4 mM, and there was no significant difference between treatments with the concentrations of 4 mM and 2 mM either.

Reports indicate that in many plant organs, aging is completely related to the decrease in the amount of polyamines and that the intracellular concentration of polyamines, especially spermidine, is high in young organs but it is reduced by the aging of the organs. Polyamines and ethylene have contradictory effects on a number of physiological processes, including the aging of tissues, fruit ripening, and the abscission of organs. There are numerous shreds of evidence that ethylene has a role of an accelerator in the aging process, the ripening, and the abscission of organs. On the other hand, studies have shown that polyamines are effective antiaging materials that delay chlorophyll destruction, membrane decay, and the increase in the activity of protease and ribonuclease enzymes. The interactions of polyamines and ethylene are both, in synthesis and in the action of each other so that the synthesis or action of one of them is prevented by another. SAM is the same precursor in the biosynthesis of polyamines and ethylene, and its consumption in each of the above pathways is irreversible. The competition between the biosynthesis of polyamines and ethylene to use SAM can justify some of their conflicting relationships in plant growth. Reports indicate that the ability of polyamines to stop ACC oxidase action can be related to the removal of superoxide radicals that are essential for the transformation of ACC to ethylene. The property of removing free radicals has a positive correlation with the number of amine groups in the polyamine molecule. This means that spermidine and spermine are more effective than putrescine and cadaverine^5^. Some reports suggest that polyamines prevent the synthesis of internal ethylene by increasing the proteins in petals and ovaries and thereby they hider the aging of tissues^12^. Some argue that polyamines prevent aging by interfering with the production of essential enzymes for the synthesis of ethylene. Free and reactive oxygen radicals cause peroxidative damage to membranes and accelerate aging. It is believed that the greatest contribution of polyamines to plants is their role in strengthening the membranes by destroying free radicals. It has been reported that polyamines have antioxidant properties and spermine is the most effective polyamine in preventing lipid peroxidation. The polyamines are bonded to the membrane phospholipids and thus prevent their peroxidation^5^. Therefore, polyamines can reduce free oxygen radicals due to their antioxidant properties and, on the other hand, they can reduce the destruction of membrane lipids and maintain membrane permeability by decreasing the activity of the lipoxygenase enzyme; as a result, flower longevity and flower quality are increased^36^.

According to the results of this study, all concentrations of polyamines increased the flower longevity per plant, and the best effect was observed at the concentration of 1 mM. Also among different types of polyamines, spermine had the greatest effect on flower longevity. As mentioned, since spermidine and spermine have more amino groups, they have a greater effect on destroying free radicals than putrescine, and thus they prevent the synthesis of ethylene, so their effect on delaying aging will be greater. As seen in the present study, spermine had the greatest effect on flower longevity per plant, followed by spermidine and putrescine, respectively. Consistent with our study, there are reports of the positive effects of polyamines on the flower longevity of other plants. For example, in a study on the effect of humic acid and putrescine on roses, the results showed that the treatment of humic acid with putrescine prolonged the vase life at all levels of putrescine (2 mM and 4 mM) application^37^. The study of the persistence of *Dianthus caryophyllus* flowers showed that polyamines (putrescine, spermidine, and spermine) increased the flower life in all concentrations, while spermidine had the highest effect at the concentrations of 1 mM and 2 mM^15^.

### Chlorophyll content

Analysis of variance results showed that the effect of polyamine sources and concentrations and their interactions on chlorophyll *a*, *b* and total chlorophyll was significant at 1% level while in carotenoids only the effect of concentrations of polyamines was significant at 1% level (Table 4).

**Table 4:** Results of variance analysis of the effect of polyamines sources and concentrations on some physiological characteristics of rose ‘Herbert Stevens’.

Changes sources	DF	Mean squares			
		Chlorophyll *a*	Chlorophyll *b*	Total chlorophyll	Carotenoids
Polyamin source	2	0.00198^†**^	0.00075^**^	0.0043^**^	0.00013^ns^
Polyamin concentration	3	0.00504^**^	0.00077^**^	0.0047^**^	0.00038^**^
Source*concentration	6	0.00448^**^	0.00049^**^	0.0052^**^	0.00019^ns^
Error	24	0.00012	0.00006	0.00014	0.00008
Cv (%)		8.94	24.46	7.64	16.55

^†^ns, * and ** respectively non-significant, significant at 5 and 1 percent.

According to the means comparison (Table 3), the use of polyamines increased chlorophyll *a*. Plants that were treated with putrescine (at the concentration of 2 mM), spermidine (at all concentrations) and spermine (at the concentration of 1 mM) were significantly different from control plants. Other treatments did not show any significant difference compared to control. The highest amount of chlorophyll *a* (0.208 mg/g FW) was observed in plants treated with 1 mM spermine, but it had no significant difference with 4 mM spermidine treatment. The lowest amount of chlorophyll *a* (0.09 mg/g FW) was also observed in control plants. Regarding chlorophyll *b*, the results of the analysis of the data (Table 3) indicated that only spermine (1 mM and 2 mM) and spermidine (4 mM) exhibited significant differences with the control, but other treatments had no significant difference with the control plants. The highest chlorophyll *b* content (0.058 mg/g FW) was observed in plants treated with 4 mM spermidine and the lowest chlorophyll *b* content (0.005 mg/g FW) was observed in the treatment of spermine at the concentration of 1 mM. But, there was no significant difference in different concentrations of putrescine treatment with control plant in chlorophyll *b* content. Finally, according to the results of the means comparison (Table 3), the application of putrescine (2 mM), spermidine (1, 2, and 4 mM) and spermine (1 mM and 2 mM) caused significant differences in total chlorophyll with control. The highest total chlorophyll content (0.247 mg/g FW) was related to spermidine treatment at the concentration of 4 mM, and the lowest one (0.119 mg/g FW) was related to spermine treatment at the concentration of 4 mM.

Polyamines are essential for cell division and proliferation in all cells and are involved in various growth processes, including the action of pigments, protein synthesis, the integration of the structure of nucleic acids, and the integration of the cell membrane^38^. It should be noted that the content of chlorophyll is affected by nutritional management, especially nitrogen management. Nitrogen deficiency can inhibit the formation of chlorophyll and cause a decrease in leaf chlorophyll content^39^. Nitrogen is a part of the enzymes involved in the synthesis of chlorophyll and is a part of the chlorophyll molecule. Therefore, the enhancement of nitrogen concentration can increase nitrogen uptake and chlorophyll content^40^. On the other hand, polyamines regulate the structure and action of membranes involved in photosynthetic processes. The application of polyamines maintains the stability of chloroplast membranes and prevents chlorophyll degradation^5^. Possibly, polyamines prevent chlorophyll degradation by inhibiting peroxidase activity^20^. Exogenous polyamines postpone the decomposition of chlorophyll in protoplasts and prevent the activity of the protease enzyme. It has been observed that in leaves harvested from many plants, the polyamines prevent the increase in RNase activity, and also the treatment of the polyamines delayed and reduced the activity of the enzyme protease and also prevented chlorophyll degradation. Probably, all these results are related to the anti-ethylene role of polyamines^5^. Due to the anti-ethylene role of polyamines and since ethylene degrades chlorophyll, polyamines, especially spermidine, prevent the production of enzymes involved in ethylene production and prevent the production of free radicals that degrade chlorophyll. Polyamines also prevent the degradation of chlorophyll by reducing the activity of hydrolytic enzymes on the thylakoid membrane^41^. Since polyamines are related to the synthesis of cytokinins and given that one of the roles of cytokinins is the evolution of chloroplast life and the postponement of leaf aging^42^, it is possible that the polyamines can help maintain the chlorophyll of the leaves in this way. Studies have shown that polyamines can increase chlorophyll content by regulating the expression of genes involved in chlorophyll synthesis^43,44^.

Therefore, as noted, since polyamines have nitrogen in their structure, and nitrogen is a part of the chlorophyll molecule, and due to the anti-ethylene role of polyamines and its effect on biosynthesis and activity of phytohormones, polyamines prevent chlorophyll degradation and increase chlorophyll content. In this study, spermidine had the greatest effect on chlorophyll content at the concentration of 4 mM. Hu *et al*.^45^, showed that under salinity-alkalinity stress, PBGD (chlorophyll biosynthesis gene porphobilinogen deaminase) expression was down-regulated and chlorophyllase expression was up-regulated, and after spermidine treatment both of them were reduced. Their results showed that exogenous spermidine had an important role in chlorophyll protection under stress by increasing chlorophyll synthesis and preventing its degradation. Results of a study on Zoysia grass, showed that the application of spermine and spermidine reduced the degradation of chlorophyll and maintained cell membrane stability. Polyamines effectively reduced ROS production by promoting antioxidant enzyme activity and then inhibiting lipid peroxidation^46^. Studies on many plants show the positive effect of polyamines on chlorophyll. In *Gladiolus grandiflorum* L., *Dahlia pinnata* L., *Salvia splendens*, and *Celosia argentea* L., putrescine application has increased the content of chlorophyll versus the control^16,17,20,47^. The results of studies on *Chrysanthemum morifolium* Ramat. and *Dianthus caryophyllus*, have shown that polyamine treatments (putrescin, spermidine, and spermine) increased the chlorophyll content compared to control^15,48^. Danaee and Abdossi^49^ also reported that foliar application of polyamine (putrescine, spermidine and spermine) resulted in a significant increment of total leaf chlorophyll content; between different concentrations of polyamine treatments, spermine (100 ppm) had the greatest effect on leaf chlorophyll content. The results of study on Fennel plant showed a significant effect of putrescine application on the chlorophyll *a*, *b* and total chlorophyll content. In this study, leaves of treated plants with 0.5 mM putrescine showed the highest chlorophyll content. But treatments with 2 mM putrescine showed the reduction in chlorophyll *a*, *b* and total chlorophyll content^50^.

### Carotenoids

**Figure 3:**
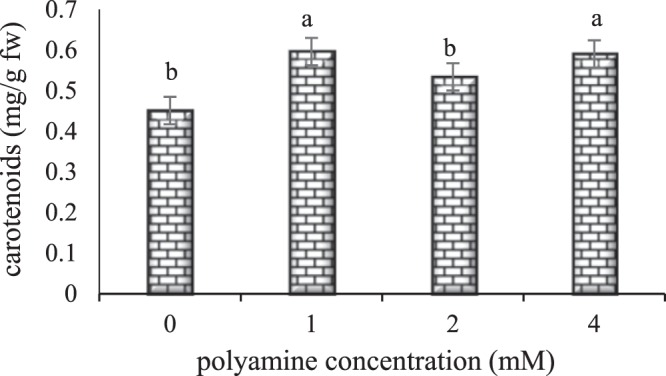
The effect of polyamine concentration on carotenoids content of rose ‘Herbert Stevens’. Values followed by the same letter(s) do not differ significantly at $\propto$ = 0.05.

The results on the effect of different concentrations of polyamines on leaf carotenoids content (Fig. 3) showed that the treatment of polyamines at the concentrations of 1 and 4 mM had a significant effect on leaf carotenoids content, but the treatment with 2 mM polyamine did not bring about any significant differences with the control. According to Fig. 3, the highest increase in leaf carotenoids (0.058 mg/g FW) was observed in treatments with the concentration of 1 mM and the lowest level of carotenoids (0.045 mg/g FW) was related to the control plants. However, no significant differences were observed between different concentrations of polyamines.

Polyamines can bind directly to the acidic groups of phospholipids on the plasma membrane bilayer, thus affecting the stability and permeability properties of the membranes^51^. In a study on Indian mustard, increasing of chlorophyll *a, b* and carotenoid levels by polyamines treatment was connected to the prevention of chlorophyll loss and preservation of thylakoid membrane structure. It seems that polyamines may exert their effect by preserving thylakoid morphology and chlorophyll levels through an interaction with the negatively charged loci in the membranes^52^. In a research on tomato fruits that expressed a high level of spermidine synthase synthesis gene, the results showed that transgenic tomatoes had greater amounts of carotenoids than wild tomatoes. Transcription-dependent analysis showed that changes in polyamine content in transgenic fruits affected stable status of gene transcription levels involved in carotenoid metabolism and led to an increase in carotenoid content. The results revealed that the genes responsible for carotenoids biosynthesis were more regulated, while the genes involved in carotenoids decomposition stopped. According to our results, the mechanism for regulating the formation of carotenoids occurs during the transcriptional phase^53^. Probably, the polyamines affect the transcriptional phase and regulate the genes responsible for the accumulation of lycopene. However, it is unlikely that transcriptional regulation is the only mechanism by which carotenoids are biosynthesis^54^. As it was observed in this study, the carotenoids content of the leaf was increased with the increase in polyamine concentration. Studies on *Calendula officinalis*, *Gladiolus grandiflorum*, *Celosia argentea*, and *Salvia splendens* have reported an ascending trend of carotenoids content with the increase in polyamines concentrations^16,17,20,35^. In a study on Fennel plant results indicated that application of putrescine exhibited significant effects on carotenoids and the highest carotenoids content was observed at treated plants with 0.5 mM putrescine^50^.

### Leaf’s macro elements (NPK)

Analysis of variance results showed that the effect of polyamine sources was significant only in potassium while the effect of polyamine concentrations on nitrogen, potassium and phosphorus was significant at 1% level (Table 5).

**Table 5:** Results of variance analysis of the effect of polyamines sources and concentrations on Leaf’s macro elements and antioxidant enzyme activities of rose ‘Herbert Stevens’.

Changes sources	DF	Mean squares					
		N	P	K	catalase	Ascorbate peroxidase	Guaiacol Peroxidase
Polyamin source	2	1622^†ns^	8.24^*^	165.65^ns^	2.68^ns^	1.21^ns^	2.77^ns^
Polyamin concentration	3	16473^**^	10.86^**^	4713.43^**^	1.56^ns^	7.15^ns^	4.21^ns^
Source*concentration	6	1686^ns^	3.43^ns^	349.22^ns^	8.44^ns^	1.26^ns^	3.79^ns^
Error	24	982	2.09	164.16	1.57	1.27	13.02
CV (%)		15.80	14.27	18.60	28.25	21.40	26.53

^†^ns, * and ** respectively non-significant, significant at 5 and 1 percent.

The results on the effect of different concentrations of polyamines on nitrogen content (Table 6) indicated that only its treatment at the concentration of 1 mM created a significant difference with control and other treatments hadn’t any significant differences with the control plants. According to Table 6, the highest amount of nitrogen (256 mg N/plant) was associated with 1 mM polyamine, and the lowest one (150 mg N/plant) was observed in plants treated with 2 mM polyamine, which had insignificant differences with the control and the treatment at the concentration of 4 mM. With respect to the effect of polyamine treatments on phosphorus content, it can be seen in Table 6 that among different types of polyamines, spermidine had the highest effect on plant phosphorus content (13.195 mg P/plant) and the lowest phosphorus content (7.73 mg P/plant) was related to the spermine 2 mM.

**Table 6:** The effect of putrescine (Put), spermidine (Spd) and spermine (Spm) on root length, root fresh and dry weight and chlorophyll content of rose ‘Herbert Stevens’

Treatment	Nitrogen (mg/plant)	phosphorus (mg/plant)	potassium (mg/plant)
Control	198.20^abcd^	9.78^ab^	36.87^b^
Put 1 mM	264.78^a^	10.183^ab^	75.66^ab^
Put 2 mM	153.97^bcd^	8.623^ab^	67.41^ab^
Put 4 mM	133.42^d^	8.79^ab^	79.26^ab^
Spd 1 mM	255.85^ab^	13.195^a^	88.80^a^
Spd 2 mM	168.80^abcd^	10.50^ab^	77.75^ab^
Spd 4 mM	219.79^abcd^	10.52^ab^	84.83^a^
Spm 1 mM	249.19^abc^	11.35^ab^	65.65^ab^
Spm 2 mM	142.65^cd^	7.73^b^	68.38^ab^
Spm 4 mM	196.27^abcd^	11.50^ab^	108.27^a^

In each column, values followed by the same letter(s) do not differ significantly at $\propto$ = 0.01.

According to means comparison (Table 6), the treatment of polyamines at different concentrations increased potassium content. According to Table 6, the highest amount of potassium (108.27 mg K/plant) in plants leaves was related to spermine 4 mM and the lowest amount of potassium (36.87 mg K/plant) was related to the control plants.

Reports indicate that polyamines interfere with the root process, and the exogenous use of polyamines improves root structure by increasing the percentage of narrow and hairy roots and reducing thick roots. These changes improve the absorption of the elements and increase their concentrations within the plants. On the other hand, polyamines can act as an additional source of nitrogen for the plants and improve their growth^55^. Putrescine has been reported to increase nitrogen uptake and storage. Nitrogen leads to the formation of nitrogen compounds such as amino acids that protect the plant from stress conditions^14^. Phosphorus is an important component of nucleic acids, phospholipids, coenzyme, NAD, NADP, and ATP. Therefore, the increase in phosphorus content after polyamine treatment can indicate the effective role of polyamines in plant growth and productivity^20^. The foliar application of polyamines increases the absorption of some elements, especially potassium. It should be noted that the critical role of potassium in photosynthesis has been demonstrated by its direct effect on growth, photosynthetic pigmentation, and carbon dioxide absorption^14^. Polyamines can increase the activity of metabolic processes in the plant, and thus the physiological activity of plants is improved by the increase in the root efficacy to absorb macronutrient nutrients from the soil^56^. Therefore, polyamines help to the better uptake of elements by plant roots through increasing photosynthesis intensity and root discharge^57^. On the other hand, it should be noted that reducing the concentration of the elements by increasing nitrogen levels is related to plant growth and dilution effects. In many cases, nitrogen increases the amount of plant dry matter and thereby it decreases the concentration of the elements in the plants^58^.

Considering the reasons mentioned above and the fact that an increase in root length was observed in the present study, it is likely that the rate of absorption of the elements has been increased. However, considering that spermidine has the greatest effect on root length and root dry weight, and because phosphorus is absorbed more from the tip of the roots, spermidine treatment has the greatest effect on the amount of plant phosphorus. It should also be noted that higher nitrogen uptake is due to the fact that polyamines contain nitrogen in their structure. Therefore, one of the main causes of nitrogen uptake of the plant is due to its presence in the structure of polyamines. Of course, this increase may also be due to increased absorption by the roots. In general, it can be stated that among different concentrations of polyamines, the treatments with the concentration of 1 mM had the greatest effect on the amount of nitrogen, phosphorus, and potassium in the plants. In general, it should be noted that the foliar application of compounds such as polyamines is more effective in the physiological processes of the plants and has an indirect and limited effect on the absorption of elements. The results of this study were in accordance with the results of studies on *Freesia refracta* (Jacquin) Klatt, *Bougainvillea glabra*, *Gladiolus grandiflorum* and *Populus euramericana*^10,20,32,55^ and the results of a study on the effect of polyamine application on *Rosa hybrida*. It was revealed that polyamine application has significant effect on leaf NPK. Results of this study showed the highest nitrogen and phosphorus concentration in 1 mM spermine treatments and a significant increase in potassium concentration in comparison with control plant^57^.

### Antioxidant enzymes activity

Based on the results of analysis of variance (Table 5), antioxidant enzymes (catalase, ascorbate peroxidase, and guaiacol peroxidase) were not affected by the polyamine treatments. Polyamine treatment had no effect on antioxidant enzymes as expected. One of the most important plant protection mechanisms in stress conditions is to prevent the production of free radicals^59^. The effects of free radicals include the decomposition of chlorophyll and protein oxide^60^. They also cause lipid peroxidation and damage to the cell membrane^46^. Polyamines are likely to exert their effects in reducing the adverse effects of stress by directly interacting with membranes, reducing oxidative activity, acting as compatible osmolytes or ionic feature^61^. It has been observed that polyamines such as putrescine, spermine and spermidine regulate the apoplastic antioxidant activity of various antioxidant enzymes. An increase in the levels of endogenous or exogenous polyamines results in increased scavenging of oxygen and hydroxyl free radicals^62^. Polyamines appear to exhibit their antioxidant effect by inducing the expression of genes that encode antioxidant enzymes. Thus, polyamines may act not only as scavengers of free radical but also as activators of the expression of antioxidant enzymes encoding genes^63^. Similar to the results of the above study, studies on different plants have also shown that exogenous polyamines did not show any significant effect on antioxidant enzyme activity of plants under non-stress conditions, but polyamine treatment in plants subjected to stress led to an increase in the plant antioxidant enzyme activities compared to untreated plants^46,52^.

## Conclusion

In general, it can be concluded that the application of polyamines can remarkably influence the growth and developmental characteristics of the plants in various ways. Among three polyamines, putrescine was most effective in root dry weight, spermidine in root length, chlorophyll content, plant phosphorus and spermine affected on root fresh weight and flower longevity. Polyamine concentration of 1 mM had the potent effect on flower longevity, carotenoids, nitrogen and phosphorus content. Polyamine treatments had no significant effect on the number of flowers per plant and antioxidant enzymes.
